# A Treatise on the Functional and Structural Changes of the Liver, in the Progress of Disease; and on the Agency of Hepatic Derangements in Producing Other Disorders; with Cases, Dissections, &c.

**Published:** 1836-04-01

**Authors:** 


### A TREATISE ON THE FUNCTIONAL AND STRUCTURAL CHANGES OF THE LIVER, IN THE PROGRESS OF DISEASE; AND ON THE AGENCY OF HEPATIC DERANGEMENT IN PRODUCING OTHER DISORDERS. With numerous Cases, exhibiting the Invasion, Symptoms, Progress, and Treatment of Hepatic Disease in India. By *W. E. E. Conwell*, M.R.I.A. Surgeon of the Madras Establishment, &c. Octavo, pp. 531. Duncan, Paternoster Row, Oct. 1835.

MR. CONWELL's work is entitled to especial attention, as exhibiting the result of 20 years' accurate observation of hepatic disorders and diseases, in a climate which, unfortunately, furnishes but too many subjects for the investigations of the physician and pathologist. The plan which the author has pursued, renders his work more particularly adapted for the young medical practitioner, when he embarks for India, than for the profession of this country generally—and for this reason, that he goes, we think, unnecessarily into the anatomy and physiology of the organ in question; and dwells with great minuteness on the modus agendi of the various causes which, in hot climates, deteriorate the structure, and derange the functions of the biliary apparatus. If we were to add, that Mr. C. has indulged pretty freely in reasoning very much allied to hypothesis—and diluted his matter with rather too many words, we would probably not be very wrong, nor very censorial. But we are far from falling into the recent mania for HYPERCRITICISM—a system which aims entirely at exhibiting the acumen of the REVIEWER, without the slightest regard for the benefit of the public, or the justice due to an author.

The work is divided into two parts—the one doctrinal—the other clinical; the latter consisting of a series of abridged cases and dissections. The first part contains five chapters, the first of which is entirely anatomical and physiological, which we must pass over. We must confess that some portions of Mr. C.'s physiology appear to us rather fanciful, and require *confirmation*, as the Americans say, when they communicate INTELLIGENCE EXTRAORDINARY. The second chapter embraces the—

### CAUSES OF HEPATIC DISORDER.

The disposition to prolixity, though apparent to the experienced eye in the first chapter, will be more conspicuous in the second—because the anatomical and physiological details, in the former, are facts ascertained, and the prolixity will be considered merely as minuteness. But, in the second chapter, the author has ventured to tread on very debateable ground, and, were we inclined to criticism, we certainly should not fear to break a spear with the author on several points of etiology. But we will keep to the sober and useful, leaving refinement and speculation for those of our contemporaries, whose literary appetites are allied to the physical GUSTO of the day—those who relish a haggis or a ragout far better than a hare or a saddle of mutton.

Among the causes of hepatic disease, our author first adduces external injuries, as falls, accidents, &c. whether on the region of the liver or the head. Secondly, he alludes to, and discusses the effects of, intemperance in drink. The *modus agendi* of vinous and spirituous potations is well sifted, and Mr. Conwell is a decided apostle of the antediluvian beverage—namely, Adam's ale—or spring water. He is not, however, a rigid disciplinarian, for he allows his patients toast-water, barley-water, and rice-water, or congee, *pro re nata.* He even rises into the generous—we had almost said the VOLUPTUARY, sometimes.

"The *habitual* use of wine is not necessary to the preservation of health in India. A moderate quantity is not *always* injurious ; and it is less so if taken at dinner, after the sun goes down. In wet, damp, or cold weather, stimuli are occasionally useful, and produce not then the irritating effects that follow their use in the hot dry weather of India." 66.

This, we call liberality ; and, upon the whole, we believe it the true doctrine of tropical hygiene. But we cannot expect that it will be generally followed in a region where the European is very often isolated from social intercourse with his countrymen—cut off from books and journals—and left to the solitary enjoyment of reflection, where the materials of reflection are thinly strewed around him. In such cases, who can wonder that the European should be tempted to the use of those exhilarating potations which elevate the spirits, although only for the moment, and solace the dull hours, days, and years, spent beneath a tropical and enervating sun !

Our author enters his caveat, also, against food of rich, nutritious quality—rich sauces, condiments, stews, &c. as tending to gorge the vessels of the portal circle, already too much disposed thereto by the high range of temperature. The effects of an Indian climate on the European constitution are thus graphically and accurately portrayed.

"Experience shows the changes ordinarily produced on European constitutions by a long residence in India. The European, after several years' residence, exchanges his clear complexion for a parchment, yellow, a leaden, or a dark, pale yellowish colour. His vascular and muscular tissues become flaccid and relaxed, and his animal and mental energies more or less diminished. His general health is impaired, and he continues, particularly as his residence becomes protracted, in a state of valetudinarianism. Medicine is at last constantly necessary to stimulate the functions of the liver, and of the gastro-intestinal tube. This is a very summary, but an exact sketch of those changes which the greatest number suffer from an inter-tropical residence. Some men retain health under singular disadvantages in India, as in all other parts ; but they are here, as elsewhere, comparatively very few. Those least exposed to the inconveniences and evils of the climate, as might be expected, suffer the least from a residence in it." 71.

That temperature alone is not the only cause of hepatic disease, is proved by the less frequency of this complaint in countries, where the range of heat is higher than in India—as in the West Indies, the coast of Guinea, &c. There is little doubt, indeed, that there is something in the climate of India which specifically induces to hepatic disorder. Mr. C. seems inclined to attribute this predisposition to the luxurious diet of the Europeans there, and supports this by the fact, that the Hindoos are not prone to hepatitis, their prevailing maladies being fevers, " depending on derangements of the gastro-intestinal tube." But, with deference to Mr. C.'s judgment, we submit that it by no means follows that the same cause shall produce the same effect in a Hindoo and a European. It is far more likely that these effects should differ, in consequence of difference of constitution, than that the causes of them should be of different kinds. Are not Europeans subject to fevers and gastro-intestinal complaints? Their robust constitutions and diet of animal food doubtless predispose them to inflammatory and congestive affection of the liver; but we do maintain that the same endemic cause which produces fevers in the Hindoo, produces hepatitis in the Englishman.

We agree with our author, that the depressing passions are more deleterious in a hot than in a temperate climate. The want of exercise is also injurious to the European—and the heat of the climate leaves him little remedy, except temperance. Our author considers that long or repeated courses of mercury tend to induce hepatic abscess. Atmospheric vicissitudes are next adduced, and long dissertations are gone into, to explain the modus agendi of this, and all the preceding morbific agents. These dissertations, we think, are generally unnecessary.

The following is the category of symptoms that indicate approaching or existing hepatic disease.

" Symptoms of Acute Hepatic Diseases. *Symptoms of Hepatitis produced by Nervous Agency.*

1. Severe, deep-seated dull pain in the posterior part, or in the centre of the head.
2. Pain and stiffness of the back of the neck and base of the head.
3. An arid state of the fauces and difficulty of correct enunciation, red tongue, loss of fur, total disappearance of the lingual papillæ.
4. Pain of the back of the neck, and of the posterior-superior part of the shoulder, from the spine along the margin of the trapezius to the shoulder-joint.
5. Strong and painful beatings of the carotids.
6. Stiffness, soreness, sense of weight or oppression in the lower part of the neck; increased action, pain, soreness, or oppression of the heart.
7. Pain at the præcordia.
8. Embarrassment of breathing, and oppression about the epigastrium, lower part of the thorax, or hepatic region.
9. Pain in the region of the stomach, loss of appetite, nausea, and various dyspeptic symptoms; thirst, febrile excitement, especially after eating; vomiting, white-furred tongue, prostration of strength.
10. Pain in one side of the face, sometimes apparently connected with the alveolar processes, and sometimes with the ear.
11. Pain in the right side, just at the gall-bladder, or in any part of a line parallel with it, but generally on the right side.
12. Pain, numbness, heaviness, or stiffness of either arm, or both.

13. Crick, or pain of the neck.
14. Stiffness and pain of the loins.
15. In the early stage, irritation, local pain, and sense of weight in the loins, with diminished excretion from the kidneys ; the urine being dark like wine lees, or the colour of brandy.
16. Pain at the top of the shoulder, or extremity of the clavicle.
17. Pain, numbness, heaviness, or stiffness of the shoulder.
18. A shooting pain in the breast.
19. A constant dry cough.
20. Dyspnœa, with sense of oppression and tightness, with or without pain.
21. A dragging or a gnawing pain about the region of the liver.
22. A stitch, catch or lancinating pain on breathing, about the lower part of the chest.
23. Pain in the region of the liver, on turning in bed, moving suddenly or quickly, or on the body being shaken by making a slip or false step ; frequently with tenderness on pressure.
24. Disturbed sleep.
25. Greatly increased action of the abdominal aorta, and frequent palpitation of the heart.
26. A sense of fulness in the chest, and inability to make a full inspiration.
27. A sense of tension, or heat, at the epigastrium.
28. Pain extending in the course of the colon, from the cæcum to the sigmoid flexure.
29. Pain of the right kidney, more rarely of the left.
30. The patient lies with more ease on the right side ; but this varies according to the extent and part affected, and to the new relations or adhesions that part may have formed.
31. Pain of the back, simulating rheumatism, from its greater severity at some than at other times.
32. A sense of strangulation at the larynx.
33. Pain in the eyeball.
34. Pain in the forehead over the eyes.
35. Pains about the pelvis and superior portion of one or both thighs.
36. Pain of the right or left knee.

*Symptoms produced by an irritated and congested State of the Lumbo-abdominal Ganglia and Plexuses, and of the Cauda Equina.*

37. Cramp of the right or left leg, or both.
38. Pain of the right or left heel.
39. Pain on the dorsum of right or left foot.
40. Cramps of the soles of the feet, and toes.
41. Numbness of the legs and feet.
42. A sense of formication on the legs and feet.
43. A burning sensation of the legs and feet, observing regular daily paroxysms that produce excruciating pain.
44. A degeneration of the tissues, whereby the muscular and cellular tissues of the legs and feet become rigid and hard." 83.

Most of our readers, we apprehend, will be inclined to doubt whether all of the above symptoms are indicative of hepatic disease. That many of them may occur in a patient labouring under hepatitis, we do not deny; but we very much doubt whether they are all referrible to the disease of the liver. At all events, there is scarcely one of them that does not occur where there is no hepatitis at all. The following code of symptomatology is less equivocal.

" *Symptoms indicated by the renal excretion* :—

1st. The urine is scanty, red, dark, tinged with bile, like wine lees, impure, opaque, and largely blended with a substance like coffee or chocolate grounds.

2d. The urine being like stale or recently prepared decoction of cinchona and opaque, then after a few days, becoming largely blended with pus, quite opaque and milky for several days; and afterwards gradually becoming like milk-whey, and opaque. As the removal of pus from the liver decreases the urine generally becomes progressively pale.

*Symptoms resulting from deranged action of the abdominal circle of vessels.* Deranged hepatic action induces engorgement of sundry parts of the abdominal circle of vessels, and according to individual condition and circumstances produces: *first,* hepatic diarrhœa; *second,* dysentery; *third,* ulcers either of the large or small intestines, or both; *fourth,* inflammation of the mucous coat either of the small or large intestines; *fifth,* engorgement of the mucous coat and piles; *sixth,* peritoneal inflammation; or, *seventh,* mesenteric disease.

*Symptoms from the general circulation.* The pulse is hard and full, large or small, and sometimes oppressed; or it is dilated, soft and feeble.

*Symptoms as to the tongue.* 1st, The surface is red, furred white, with thirst; 2d, furless and red, sometimes smooth; 3d, marked with sulci and red; 4th, presenting a pale spot near the top; 5th, ulcerated and red; 6th, dark brown, dry and hard.

*Physical symptoms.* Fulness, tenseness, or hardness of the region of the liver, and sometimes distinct swelling or dilatation of the inferior part of the thorax, and occasionally puffiness of the integuments over the liver.

*Symptoms of the gastro-intestinal tube.* 1st, In the early stage, costiveness and rarely looseness; 2d, in chronic hepatic disease, diarrhœa and dysentery occur.

*Symptoms indicated by the skin.* In the early or acute stage, stinging heat and dryness. In the chronic stage, or after suppuration, moist or wet skin from profuse sweats.

*Symptoms indicated by the palms of the hands and soles of the feet.* Always in the early stage, and sometimes in the chronic, burning arid heat, especially after meals, with fulness and hardness." 85.

The terminations of hepatitis are in resolution, effusion, adhesion, suppuration, ulceration, granulation, cicatrization, mortification, metastasis—and lastly, chronic disease. To which he has added—degeneration into other tissues. "The *effusion* into the hepatic tissue is met with in Europe, as shewn by the existence of large cysts and hydatids." Some people will consider this vague pathology. Hydatids are generally thought to be organized living beings—and the mode of accounting for their formation by mere serous effusion into the tissues of the liver, is anything but satisfactory. Our author has only seen one instance of mortification in the substance of the liver.

" *Metastasis,* or transition of inflammation from the liver to other parts, is of frequent occurrence. The gastro-intestinal tube, and especially its mucous lining, the kidneys, spleen, colon, peritoneum, the lungs, pleura, and the external surface of the body, are all of them subject to inflammatory attacks, translated or extending from the liver." 90.

The foregoing are the principal points, of a practical nature, which we have been able to select from the second chapter of the work.

The third chapter is on " functional disorders of the liver." It is, however, so divided and subdivided, that we have given up, in despair, any attempt to lay before our readers any idea of its contents. Verily Mr. C. has " cut blocks with a razor." From the fourth chapter we can only make one extract, embracing the symptomatology and pathology of "congestive nervo-bilious fever, as seen in the East Indies."

"After slight chills or rigors (in some cases only discovered by inquiry,) febrile heat occurs with partial and irregular remissions. The pulse is soft, not large, from 98 to 130. The tongue is at first but little affected; it afterwards successively becomes clammy, furred, then red, sulcated, and ultimately dark and dry. The skin is at times hot; the hands and feet cold, and in other cases the surface is cold with profuse sweats; the head is always hot. The cold surface or extremities with occasional fainting or convulsive fits at the commencement, is diagnostic of this disease in its severe form. When the surface is cold with profuse sweats, the skin does not turn yellow; nor does it in every case where there is heat, but yellowness occurs only in those latter cases. Pain of the head is never wanting, and the patient often says. ''tis heavy as if filled with lead:' there is a sense of burning heat at the epigastrium, with constant retching, vomiting, and thirst. As in cholera, when the skin is cold and pouring forth cold sweat from every pore, so here, when the skin is precisely in the same state, the patient complains of burning and heat of the surface, and force alone will prevent him from throwing off the bed-clothes. There are intense restlessness and jactitation, the conjunctiva is injected, and the eye, from bright, soon becomes heavy like that of a person who has taken a quantity of wine. The bowels are always costive, especially in the earlier stages. The putrid symptoms, that mark the disease in the West Indies, &c. do not occur in the East.

*Pathology.* It appears, as least from my dissections, and from a physiological analysis of the symptoms, that this fever is formed by an association of several morbid states. 1st, Inflammation of the interior surface of the arteries. 2dly, Cerebral congestion. 3dly, Congestion and irritation of the envelopes of the ganglionic system. 4thly. Congestion and irritation of the inferior twenty-four inches of mucous membrane lining the ilium. 5thly, Gaseous distension of the ascending and right arch of the colon that presses on, displaces, and folds the stomach to the left. 6thly, Hepatic congestion. 7thly, In some countries, a putrid state of the fluids. The inflammation of the arteries, and congestion of the cerebrum, of the liver, ganglionic system, and the lower part of the ilium (ileum), are all branches from the same stem,—that is, the congestion of the ganglionic system taking place, it follows that the movements of the heart, and the circulation in the capillary vessels, should become deranged, because they are under the control of those nerves. The very intimate connexion between the pulmonary and cardiac plexuses, and their extension to the stomach and solar plexus, explain why malaria, acting on the lungs, exerts an immediate influence upon the pulmonary, cardiac, gastric, and abdominal plexuses" 190.

"*Treatment.* In the outset, the head should be shaved, as extensive advantage results from keeping the head cold, which cannot otherwise be so well effected. The rapid tendency of the disease to terminate in cerebral engorgement and death, warrants the prompt employment of the most energetic remedies. If general bleeding has been omitted at first, its subsequent use hastens death; after the first stage, or twenty-four hours, leeches should be largely used. In the case of an athletic European, thirty leeches should be applied around the base of the head and over the sutures, fifteen down the spine, ten to the epigastrium, and five to the anus. Blisters should then be used—one between the shoulders and one to the epigastrium, the second to be applied when the first is removed. A free dose of calomel ought also to be given, once or twice a day, with or without opium. Half the above number of leeches should be repeated after ten hours, if the pulse, or febrile heat, indicate their use, and strong sinapisms applied to the lower extremities, if cerebral engorgement threatens delirium or coma. In milder cases of congestion, hot mustard baths to the lower extremities are useful. Cold applications to the head, after shaving it in the earliest stage, should never be omitted. General cold affusion is very seldom required in this disease. Mercurial purgatives cannot, with safety, be omitted. The danger subsides when ptyalism occurs, but, unless depletion enables the vascular and absorbent systems to act freely, mercurials will not produce this effect.

When a perfect remission occurs, and warrants the use of quinine, it should be used, whether ptyalism has occurred or not." 195.

We must pass over some observations on acute hepatitis, which are not important, to notice—

### CHRONIC HEPATITIS.

After acute hepatitis, congestion, or obstruction, a valetudinary state often takes place, marked by debility and dyspepsy.

"In this condition of disease, the hepatic function is depraved; there are many obscure and some distinct symptoms of hepatitis; and the gastro-intestinal functions rarely continue in a natural state for more than a few days without treatment." 201.

"*Symptoms and Pathology.* The symptoms vary with the stage of the disease, and the pathological condition. Acute hepatitis inefficiently or improperly treated usually terminates in suppuration, one or more abscesses forming. These abscesses may be large or small; they may occupy parts near the convex surface or the centre of the gland, or the inferior surface, the extreme right or the left, or the anterior or the posterior margin of the viscus. If they occupy the centre, they will be announced by hectic fever and sweats, with tendency to diarrhœa and dysentery, accompanied by great prostration of strength and dyspepsy. Pains simulating rheumatism are occasional concomitants when the absceses occupy parts adjoining the inferior surface; because the solar and lumbo-abdominal plexuses and ganglia are then more frequently affected in consequence." 202.

"When abscesses are placed in the centre of the gland, the pus either passes through the hepatic veins, or remains and excites occasional or partial accessions of inflammation and fever; the original cyst being enlarged by ulceration and suppuration, which advance in some cases slowly, in others rapidly."

"The soft hepatic enlargement, decrease of pain, accession of hectic and profuse sweats, sleeplessness, soft pulse, red beef-steak tongue and general debility, evince the existence of hepatic abscess." 203.

In the treatment laid down by Mr. Conwell, there is nothing particularly novel. General or local depletion, according to circumstances—counter-irritation—purgation—alterative doses of mercury—even to ptyalism, if necessary—neutral saline medicine, &c.

In the fifth chapter, the author takes "a physiological review of the principal remedies used in the treatment of hepatic diseases." But we cannot indulge even in a single extract, since the matter is more adapted for lectures from an Indian chair at Edinburgh to an audience of young gentlemen about to embark, after the curriculum of their studies was concluded, for the burning shores of the Eastern hemisphere.

Nearly half of the volume (p. 255 to p. 331) is a mere copy of the case-book, abridged—to which we must refer our readers, if any of them should have time or inclination for the Herculean task!

In conclusion, we must say that we have been greatly disappointed in the perusal of this work. We have endeavoured to give some idea of its contents to our readers; but the mode in which it is arranged and composed defies analysis.

